# Free intraabdominal catheter management post-VP shunt disconnection in pediatric patients: systematic review

**DOI:** 10.1007/s00381-025-06898-y

**Published:** 2025-07-22

**Authors:** Nazeer Aboud, Lydia Karamani, Sergio Alexander Calero Martinez

**Affiliations:** Department of Neurosurgery, Jena University Hospital, Friedrich-Schiller-University Jena, Jena, Germany

**Keywords:** Ventriculoperitoneal shunt, Pediatric hydrocephalus, Catheter disconnection, Laparoscopy, Systematic review, Shunt complications

## Abstract

Ventriculoperitoneal (VP) shunting is the standard treatment for pediatric hydrocephalus but carries a high complication rate, including mechanical failure. Distal catheter disconnection and intraabdominal migration present a unique clinical dilemma: whether to retrieve or observe the retained catheter. This systematic review evaluates reported management strategies and outcomes associated with free intraabdominal catheter fragments post-VP shunt disconnection in pediatric patients. A comprehensive literature search was conducted across PubMed, Ovid, Scopus, Embase, and Google Scholar using predefined terms. A total of 4721 unique records were screened; 31 full texts were reviewed, and 11 articles met inclusion criteria. Eligible studies involved pediatric patients with disconnected VP shunts and retained peritoneal catheters, with management details and clinical outcomes. Data extraction and NIH-based quality assessment were independently performed by two reviewers. The final analysis included 11 studies (six case series, five case reports) involving 36 patients. Laparoscopic retrieval was the predominant technique (in 10 of 11 studies), often enabling concurrent catheter replacement. Outcomes were consistently favorable, with no major complications and minimal postoperative morbidity. One study required conversion to laparotomy due to visceral adhesion. Conservative management was rarely employed and not supported by outcome data. Quality assessment rated most studies as good. Free intraabdominal VP shunt catheters in pediatric patients should be retrieved when safely feasible. Laparoscopic management appears highly effective and low risk. Despite limited high-level evidence, the literature supports early elective removal to prevent delayed complications.

**Supplementary information:**

The online version contains supplementary material available at 10.1007/s00381-025-06898-y.

## Introduction

Ventriculoperitoneal (VP) shunting remains the cornerstone of hydrocephalus management in the pediatric population. Indicated primarily for obstructive and communicating hydrocephalus, VP shunts serve to divert cerebrospinal fluid (CSF) from the cerebral ventricles to the peritoneal cavity, thereby alleviating intracranial hypertension and its associated sequelae. Hydrocephalus affects approximately 1 in 1000 live births, and while treatment with VP shunts has significantly improved outcomes, the long-term management is fraught with complications that necessitate close monitoring and often multiple revisions throughout childhood [1, 2].

Complication rates for VP shunts remain high, with overall failure rates approaching 30–40% within the first year of placement and revision rates increasing with time [3]. Among these, shunt disconnection—a mechanical failure of the system—is a notable cause of malfunction, reported in up to 7–10% of cases [4]. Disconnection most commonly occurs at the junction of the valve and distal catheter, often resulting in the migration of the free peritoneal catheter into the abdominal cavity [5].

Intraabdominal complications resulting from the presence of disconnected or migrated catheters are rare but potentially serious [6]. Reported complications include bowel obstruction, volvulus, formation of abdominal pseudocysts, knotting of the catheter, and even organ perforation with anal extrusion [7–12]. Though infrequent, these complications may present acutely and can mimic other causes of surgical abdomen in children, with some necessitating urgent intervention [9, 13].

The clinical management of a free intraperitoneal catheter following VP shunt disconnection remains controversial. Some authors advocate for prophylactic removal of the retained catheter due to the theoretical risk of delayed intraabdominal sequelae, while others propose a conservative approach in asymptomatic patients to avoid unnecessary surgical risks [14]. Various retrieval techniques have been described in the literature, including open laparotomy, laparoscopic retrieval, and percutaneous methods, each with their own risk–benefit profiles [15, 16].

Given the paucity of comprehensive guidance and the lack of consensus regarding the necessity, timing, and technique of catheter retrieval, this systematic review aims to synthesize the existing literature on the management of free intraabdominal catheters following VP shunt disconnection in the pediatric population. By identifying the frequency of associated complications and comparing management strategies, we aim to update clinical decision-making and improve outcomes in this vulnerable group.

## Methods

### Search strategy

A comprehensive literature search was conducted to identify all relevant studies addressing the management of free intraabdominal catheters following ventriculoperitoneal (VP) shunt disconnection in pediatric patients. The following databases were searched: PubMed (*n* = 105), Ovid (*n* = 56), Scopus (*n* = 130), Embase (*n* = 0), and Google Scholar (*n* = 4,430). The search included articles published up to February the 28th, 2025, using a combination of Medical Subject Headings (MeSH) and free-text terms: (“ventriculoperitoneal shunt” OR “VP shunt”) AND (“disconnection” OR “fracture” OR “migration”) AND (“intraabdominal” OR “peritoneal”) AND (“removal” OR “retrieval” OR “management” OR “laparoscopic” OR “observation” OR “surgery”) AND (“pediatric” OR “children” OR “infant”). Additional manual reference checks of relevant articles were also performed. This review follows the guidelines established by Preferred Reporting Items for Systematic Reviews and Meta-Analyses (PRISMA).

### Eligibility criteria

Inclusion criteria:

- Pediatric patients (0–18 years) with VP shunt disconnection and retained intraabdominal catheter
- Studies reporting clinical management (surgical or conservative)
- Articles presenting patient outcomes or procedural details
- Original case reports, case series, or retrospective analyses
- Reported outcomes including complications, procedural success, and follow-up

Exclusion criteria:

- Adult-only studies
- Non-English articles without accessible full-text English translation
- Reviews, editorials, or abstract-only publications
- Non-human studies
- Isolated proximal shunt issues

### Study selection

**Fig. 1:**
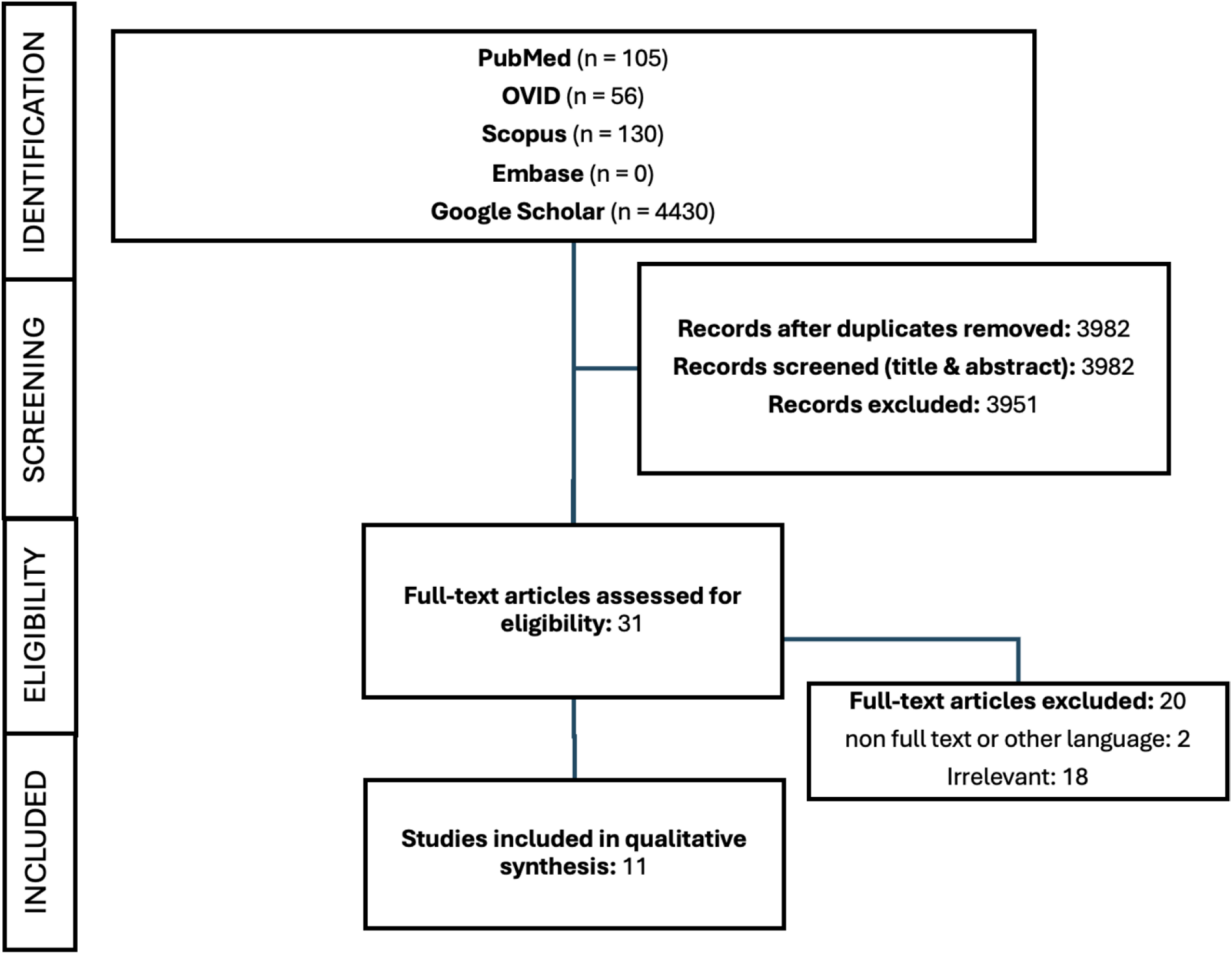
PRISMA flow chart The flowchart describes the process for the systematic review following the PRISMA convention

After removal of duplicates, titles and abstracts of 4721 articles were screened (NA, LK). Thirty-one studies were selected for full-text review, of which 11 met the inclusion criteria and were included in the final analysis (NA, LK, SACM). Disagreements were resolved through discussion or third-party adjudication. The PRISMA flow diagram summarizes the study selection process (Fig. 1).

### Data extraction

Data from selected studies were extracted using a standardized sheet (Table 1). Extracted variables included study design, number of cases, patient demographics, catheter location, removal method, complications, outcomes, and recommendations.

**Table 1:** Summary of included studies on intraabdominal catheter removal post-VP shunt disconnection

Study	Study design	Number of cases	Surgical approach	Outcomes
Davis & Wah, 1989	Case series	5	Laparoscopic	Successful removal using adult laparoscopy in children
Almetaher et al., 2018	Case series	4	Laparoscopic	Removal and revision, all cases managed laparoscopically
Deinsberger et al., 1995	Case report	1	Laparoscopic	Free catheter retrieved laparoscopically in neonate
Guzinski et al., 1982	Case series	4	Laparoscopic	Successful use of adult laparoscopic tools in pediatrics
Jackson et al., 2002	Case report	1	Laparoscopic	Single-incision retrieval and reinsertion
Kaplan et al., 2007	Case series	2	Laparoscopic/laparotomy	One case laparoscopic, one required laparotomy due to adhesions
Pierangeli et al., 1999	Case report	1	Laparoscopic	Two catheters removed, existing shunt preserved
Pomeranz et al., 1988	Case series	9	Blind palpation	No complications with blind palpation removal
Jibia et al., 2022	Case report	1	Laparoscopic	Successful bi-disciplinary retrieval with revision
Schrenk et al., 1994	Case report	1	Laparoscopic	Successful removal in both cases, one minor complication
Short et al., 2009	Case series	7	Laparoscopic	Feasibility of day-case laparoscopic retrieval confirmed

This table summarizes the included studies in this systematic review

### Quality assessment

The methodological quality of included studies was assessed using the NIH tool for case series and retrospective reviews (supp. table 1) [17]. Quality assessments were performed independently by two reviewers (NA and SACM).

## Results

### Study characteristics

A total of 11 studies were included, comprising 38 pediatric patients. Study designs included five case reports and six case series. All patients had a retained intraperitoneal catheter following VP shunt disconnection and underwent either laparoscopic or manual retrieval.

### Surgical approaches

Laparoscopy was the most common approach, reported in 10 of 11 studies. One study described blind palpation through a small abdominal incision during revision surgery [18]. Laparoscopic methods varied from single-incision to dual-port techniques, with some allowing simultaneous reinsertion of new shunt components [19].

In one study, conversion to open laparotomy was necessary due to catheter adhesion to abdominal viscera [20]. Similarly, Short et al. encountered mesocolonic penetration in one case, requiring conversion [21]. These underscore the importance of direct visualization during retrieval.

### Clinical outcomes

All laparoscopic procedures achieved successful retrieval. Reported benefits included minimal invasiveness, reduced operative times (as low as 10 min), and in several cases, same-day discharge [21]. No major complications were reported in the majority of cases, with only one minor complication noted [22]. No postoperative infections or catheter-related reinterventions were documented in follow-up.

### Timing and indications for removal

Some authors advocated for elective removal even in asymptomatic patients, citing the risk of delayed complications such as volvulus, bowel obstruction, adhesion formation, and rare but documented organ perforations. While data remains limited, the cumulative evidence suggests that retrieval of freely floating intraperitoneal catheters, when safely possible, is justified—even prophylactically [21, 23, 24].

## Discussion

Ventriculoperitoneal shunting remains the primary treatment modality for hydrocephalus in pediatric patients. Despite technological advancements and surgical refinements, shunt malfunction and disconnection are not uncommon, with disconnection rates reported between 7 and 10% [4, 25]. When a disconnection occurs, the distal catheter may remain freely within the peritoneal cavity, posing a unique management challenge. This systematic review aimed to synthesize current evidence regarding the management of these free intraabdominal catheters, focusing on safety, procedural efficacy, and outcomes. The literature consistently supports that retrieval of these catheters—when feasible—is safe and effective.

The potential for serious intraabdominal complications forms the rationale for considering removal. Documented risks include bowel volvulus [11], knot formation [10], bowel obstruction [8], perforation [9], and pseudocyst formation. While the true incidence of such events remains low, their potential severity underscores the importance of vigilance. Additionally, there is theoretical concern that retained foreign material could promote adhesion formation, chronic inflammation, or infection. Given the long lifespan of pediatric patients with hydrocephalus, even low-probability risks may warrant proactive management.

Among the studies included in this systematic review (with 36 cases), all described successful retrieval of catheters either via laparoscopy or blind palpation [18]. No major complications were directly attributable to the removal procedures themselves. Laparoscopy was the preferred approach in four studies. It offers full visualization of the peritoneal cavity and facilitates retrieval even of migrated or coiled catheters. It also allows simultaneous inspection of shunt function, assessment of peritoneal adhesions, and even reinsertion of a new distal catheter. Guzinski et al. demonstrated that adult laparoscopic tools could be safely used in pediatric patients, expanding access to this technique in resource-limited settings [15]. The single study employing blind palpation during revision surgery also reported complete removal without complication [18]. While less commonly utilized today, this technique remains valuable in cases where laparoscopy is unavailable or contraindicated. We summarize important aspects of both techniques in Table 2.

**Table 2:** Comparison between laparoscopic and palpation techniques

Feature	Laparoscopic retrieval	Blind palpation retrieval
Visualization	Direct intraabdominal visualization	No visualization; relies on manual palpation
Precision	High accuracy in locating catheter	Lower precision; dependent on surgeon’s tactile feedback
Access	Requires general anesthesia and laparoscopic equipment	Can be performed during open shunt revision, through old access
Operative time	Generally short (≤ 15–30 min)	Short (but variable depending on success)
Complication risk	Low; may include visceral adhesions or need for conversion	Very low; minimal incision and tissue manipulation
Additional interventions	Allows simultaneous shunt revision or catheter reinsertion	Limited to removal; revision requires separate step
Postoperative recovery	Quick; often same-day discharge possible	Quick if uncomplicated
Indicated when	Free catheter suspected or confirmed; full peritoneal access needed	Catheter is superficial or easily palpable during planned revision
Equipment needed	Laparoscopic tower, ports, instruments	Standard surgical set
Suitability	Preferred where resources and expertise exist	Useful in resource-limited or urgent settings

This table compares both techniques for removal of the abdominal catheter

Regarding the timing of intervention, authors diverged in their recommendations. Several authors advocated prompt removal to reduce the risk of an acute abdomen [14, 15], whereas Pomeranz et al. recommended combining retrieval with planned shunt revision [18]. This variability likely reflects differences in patient presentation, resource availability, and institutional protocols. From a risk management perspective, timely elective removal, especially when combined with shunt revision, may offer an optimal balance between risk and benefit. However, in asymptomatic patients with high surgical risk, conservative management could still be considered with close monitoring.

The limitation of this systematic review is the low quantity and quality of available literature. Despite consistency in procedural success, there remains no standardized algorithm for management. The current evidence is primarily derived from case series and retrospective reviews, lacking randomized or prospective comparative data. This makes it difficult to determine whether proactive removal improves long-term outcomes or reduces complication rates compared to observation.

Future research should focus on larger, multicenter cohort studies with prospective follow-up, comparing outcomes between removal and non-removal groups. Registries tracking long-term complications from retained catheters could also help clarify the natural history and guide evidence-based protocols.

## Addendum

During the systematic review process, two additional articles relevant to the topic were identified but could not be included in the detailed analysis due to language barriers. One article was published in Turkish [26] and the other in Japanese [27]. However, both included English-language abstracts that clearly reported successful laparoscopic retrieval of intraperitoneal catheters following ventriculoperitoneal shunt disconnection, without reported complications. These findings reinforce the argument that retrieval of a free intraperitoneal catheter should always be considered when safely feasible, especially in light of the potential long-term complications associated with leaving catheters in situ. This rationale aligns with the commentary provided in the technical note by Pomeranz et al. [18], who emphasize the importance of proactive removal when possible.

## Supplementary information

Below is the link to the electronic supplementary material.

ESM 1 (DOCX 28.8 KB)

## Data Availability

No datasets were generated or analysed during the current study.

## Declarations

This is a systematic review, so neither ethics committee nor ethical approval was required.
