# Supplementary material for: Free intraabdominal catheter management post-VP shunt disconnection in pediatric patients: systematic review
Source: Childs Nerv Syst. 2025 Jul 22;41(1):239. doi: 10.1007/s00381-025-06898-y (PMC12283846; doi:10.1007/s00381-025-06898-y)
Supplement: Supplementary file 1 — (DOCX 28.8 KB) [file 381_2025_6898_MOESM1_ESM.docx]

**Supp. Table 1. NIH Quality Assessment of Included Studies**

| Study | Clearly described objective | Consecutive cases | Comparable subjects | Clearly described intervention | Outcome measures defined | Adequate follow-up | Overall quality |
| --- | --- | --- | --- | --- | --- | --- | --- |
| Davis & Wah, 1989 | **Yes** | **Yes** | **Yes** | **Yes** | **Yes** | **Yes** | **Good** |
| Almetaher et al., 2018 | **Yes** | **Yes** | **Yes** | **Yes** | **Yes** | **Yes** | **Good** |
| Deinsberger et al., 1995 | **Yes** | **N/A** | **Yes** | **Yes** | **Yes** | **Yes** | **Good** |
| Guzinski et al., 1982 | **Yes** | **Yes** | **Yes** | **Yes** | **Yes** | **Unclear** | **Fair** |
| Jackson et al., 2002 | **Yes** | **N/A** | **Yes** | **Yes** | **Yes** | **Yes** | **Good** |
| Kaplan et al., 2007 | **Yes** | **Yes** | **Yes** | **Yes** | **Yes** | **Yes** | **Good** |
| Pierangeli et al., 1999 | **Yes** | **N/A** | **Yes** | **Yes** | **Yes** | **Yes** | **Good** |
| Pomeranz et al., 1988 | **Yes** | **Yes** | **Yes** | **Yes** | **Yes** | **Yes** | **Good** |
| Jibia et al., 2022 | **Yes** | **N/A** | **Yes** | **Yes** | **Yes** | **Yes** | **Good** |
| Schrenk et al., 1994 | **Yes** | **N/A** | **Yes** | **Yes** | **Yes** | **Yes** | **Good** |
| Short et al., 2009 | **Yes** | **Yes** | **Yes** | **Yes** | **Yes** | **Yes** | **Good** |

Supp. Table 1. It uses the NIH check list to assess the quality of the included studies in this systematic review.
